# Indirect effects of the COVID-19 pandemic on risk of gestational diabetes and factors contributing to increased risk in a multiethnic population: a retrospective cohort study

**DOI:** 10.1186/s12884-023-05659-6

**Published:** 2023-05-12

**Authors:** Yoon Ji Jina Rhou, James Elhindi, Sarah J. Melov, N. Wah Cheung, Dharmintra Pasupathy, the Western Sydney COVID-19 Pregnancy Study Group

**Affiliations:** 1 Reproduction and Perinatal Centre, Faculty of Medicine and Health, The University of Sydney, Sydney, NSW 2006 Australia; 2 Department of Diabetes and Endocrinology, Westmead Hospital, Sydney, NSW 2145 Australia; 3 Women’s and Newborn Health, Westmead Hospital, Sydney, NSW 2145 Australia

**Keywords:** Gestational diabetes, Pandemic, COVID-19, Lockdown, Gestational weight gain, Pregnancy outcome

## Abstract

**Background:**

The COVID-19 pandemic has had indirect effects on pregnancy outcomes. There is limited data on the impact on gestational diabetes (GDM) in diverse populations and the possible underlying mediators. This study aimed to assess the risk of GDM pre-COVID-19 and in two distinct pandemic exposure periods, and to determine the potential factors contributing to increased risk in a multiethnic population.

**Methods:**

A multicentre, retrospective cohort study was performed of women with singleton pregnancy receiving antenatal care at three hospitals two years pre-COVID-19 (January 2018 – January 2020), first year of COVID-19 with limited pandemic-mitigating restrictions (February 2020 – January 2021) and second year of COVID-19 with stringent restrictions (February 2021 – January 2022). Baseline maternal characteristics and gestational weight gain (GWG) were compared between cohorts. The primary outcome was GDM, assessed using univariate and multivariate generalised estimating equations models.

**Results:**

28,207 pregnancies met the inclusion criteria, 14,663 pregnancies two years pre-COVID-19, 6,890 in COVID-19 Year 1 and 6,654 in COVID-19 Year 2. Maternal age increased across exposure periods (30.7 ± 5.0 years pre-COVID-19 vs 31.0 ± 5.0 years COVID-19 Year 1 vs 31.3 ± 5 years COVID-19 Year 2; *p* < 0.001). There were increases in pre-pregnancy body mass index (BMI) (25.5 ± 5.7 kg/m^2^ vs 25.7 ± 5.6 kg/m^2^ vs 26.1 ± 5.7 kg/m^2^; *p* < 0.001), proportion who were obese (17.5% vs 18.1% vs 20.7%; *p* < 0.001) and proportion with other traditional risk factors for GDM including South Asian ethnicity and prior history of GDM. Rate of GWG and proportion exceeding recommended GWG increased with pandemic exposure (64.3% vs 66.0% vs 66.6%; *p* = 0.009). GDM diagnosis increased across exposure periods (21.2% vs 22.9% vs 24.8%; *p* < 0.001). Both pandemic exposure periods were associated with increased risk of GDM on univariate analysis, only COVID-19 Year 2 remaining significantly associated after adjusting for maternal baseline characteristics and GWG (OR 1.17 [1.06, 1.28], *p* = 0.01).

**Conclusions:**

Diagnosis of GDM increased with pandemic exposure. Progressive sociodemographic changes and greater GWG may have contributed to increased risk. However, exposure to the second year of COVID-19 remained independently associated with GDM after adjusting for shifts in maternal characteristics and GWG.

**Supplementary Information:**

The online version contains supplementary material available at 10.1186/s12884-023-05659-6.

## Background

The COVID-19 pandemic has been associated with adverse maternal and perinatal outcomes, including observations of an increased risk of gestational diabetes (GDM) [1–3]. Proposed contributors include maternal COVID-19 infection, disruption to healthcare service delivery and access, and indirect impact of wider societal changes [4].

Although early research raised concerns for direct consequences of maternal COVID-19 infection on pregnancy outcomes, more recent studies have attempted to assess the indirect effects of pandemics, particularly by evaluating populations experiencing low COVID-19 case burden but exposed to pandemic-mitigating restrictions of varying intensity. However, most of these have focused on perinatal outcomes such as preterm birth [5–10].

The mechanisms underlying the association between pandemic exposure and increased risk of GDM remain unclear, with few studies investigating GDM as the primary outcome and particularly limited data on populations with low COVID-19 case burden. Analysis of specific factors that may mediate the risk, such as the potential effects of pandemic-mitigating measures on population sociodemographic profiles or pre-pregnancy and gestational weight gain (GWG), is scarce. Furthermore, most previous studies have comprised majority-Caucasian or East Asian cohorts, impacting their generalisability to ethnically diverse populations [1, 3].

The Western Sydney Local Health District (WSLHD) is a network of hospitals in Sydney, Australia servicing a catchment population of over 1 million people and providing antenatal care for > 10,000 births per year [11]. The population is ethnically and socioeconomically diverse and the majority of pregnant women report a country of birth outside of Australia [10–12]. The population experienced changes in healthcare service delivery from the start of the COVID-19 pandemic in March 2020 but was initially exposed to low COVID-19 case burden and other than international border restrictions, limited duration of measures targeting community activity [13, 14]. Our group previously reported that the first year of the pandemic was associated with changes in sociodemographic characteristics and birth outcomes, with potential contribution of changes in antenatal service delivery [10]. Community restrictions escalated during the second year of COVID-19, with highly stringent pandemic-mitigating measures for residents of this health district in particular, including stay at home orders, exercise limited to one hour per day and essential movements prohibited to a 5 km radius at their peak. Although there was an increase in COVID-19 cases in the second year of the pandemic, maternal COVID-19 infection rates remained low [13, 15].

The pre-pandemic period and the first and second years of COVID-19 in this population thus present a unique opportunity to evaluate the changes in maternal characteristics and GWG with escalation in intensity and duration of pandemic-mitigating measures, and the potential effects of these changes on the risk of GDM in a diverse population.

## Methods

### Study population

A retrospective, multicentre cohort study of singleton pregnancies receiving antenatal care in the WSLHD, Sydney, Australia from 1^st^ January 2018 to 31^st^ January 2022 was performed, encompassing three hospitals including a tertiary referral center. The study period was defined as two years pre-COVID-19 (representing the unexposed cohort): 1^st^ January 2018 to 31^st^ January 2020, COVID-19 Year 1 (cohort exposed to short pandemic duration and brief, lower stringency restrictions): 1^st^ February 2020 to 31^st^ January 2021, and COVID-19 Year 2 (cohort exposed to longer pandemic duration and prolonged, higher stringency restrictions): 1^st^ February 2021 to 31^st^ January 2022. This study was approved by the WSLHD Human Research Ethics Committee.

Women were included if they had undergone a 75 g oral glucose tolerance test (OGTT). Multiple pregnancy, pregestational diabetes and miscarriage at < 20 weeks’ gestation were excluded.

### Pandemic-mitigating measures

COVID-19-mitigating measures were introduced in March 2020. Healthcare service delivery-related changes were in place from the start of the pandemic and persisted for the district’s maternity services throughout the study period. As previously described, these included visitor restrictions, utilisation of obstetric telehealth reviews for women with COVID-19 infection or close contacts and adoption of routine telehealth delivery of dietetic (routinely offered to overweight and obese women at booking visit) and diabetes in pregnancy services [10].

International border restrictions were introduced early in the pandemic and persisted for all of the study period. Other community restrictions varied in intensity (Supplementary Table 1), including a brief 6-week general lockdown in March 2020 with subsequent return to near-normal community activities during the first year of COVID-19. There was re-escalation of community measures in the second year, culminating in a 4-month general lockdown in June 2021. The catchment population was subject to particularly stringent community restrictions during this timeframe. The stay-at-home orders were exempted for medical appointments and investigations. The COVID-19 Year 1 cohort was thus exposed to healthcare service delivery-related changes but limited community restrictions. The COVID-19 Year 2 cohort was exposed to continuation of modified healthcare service delivery and prolonged and high intensity community restrictions [13].

### Data and outcomes

All singleton pregnancies were identified and routinely collected sociodemographic, medical, obstetric and administrative data were extracted from electronic maternity health record systems. Medical data included risk factors for GDM such as prior history of GDM, first degree family history of diabetes and history of polycystic ovarian syndrome (PCOS). Ethnicity was routinely collected but if missing, was assumed from country of birth if country not ethnically diverse, and mixed race was classified as "other". Measure of socioeconomic disadvantage was determined by correlating residential suburb at booking with the Index of Relative Socio-Economic Disadvantage of the Socio-Economic Indexes for Areas (SEIFA) 2016 from Australian census information [16].

COVID-19 infection status during pregnancy was determined from a separate prospectively collected database of COVID-19 infections. Universal 75 g oral glucose tolerance testing (OGTT) was recommended at < 24 weeks’ gestation if prior history of GDM or body mass index (BMI) ≥ 30 kg/m^2^ and at 24–28 weeks for all others and if early OGTT did not meet criteria for GDM. Modified recommendations for diagnostic testing for GDM during the COVID-19 pandemic were not adopted by our maternity services except for a brief period in the first year of the pandemic [17]. Performance of OGTT and corresponding glucose results were routinely recorded and also cross-checked with results systems of internal pathology and the most commonly used external pathology service. Pre-pregnancy BMI was determined from self-reported pre-pregnancy weight and height measured at booking visit. Pre-pregnancy BMI was categorised as underweight, healthy weight, overweight or obese in accordance with the World Health Organization (WHO) classifications [18]. Weights were measured at each in-person antenatal visit. Rate of first and second trimester GWG was determined by comparing pre-pregnancy weight with weight measurements up to 27 weeks and 6 days’ gestation. Excessive second trimester GWG was determined using weight measurements from 14 to 27 weeks and 6 days’ gestation in accordance with the Institute of Medicine and National Research Council (IOM) recommendations [19]. GWG in the third trimester was not analysed as these weights were measured post-GDM diagnosis.

The primary outcome was GDM, defined by 75 g OGTT results in accordance with the International Association of the Diabetes and Pregnancy Study Groups (IADPSG) criteria: one or more of fasting ≥ 5.1 mmol/L, 1-h glucose ≥ 10.0 mmol/L or 2-h glucose ≥ 8.5 mmol/L [20].

### Statistical analysis

Statistical analyses were performed using Stata Special Edition Version 14.2 (StataCorp LLC, College Station, TX, USA). All hypotheses were examined at a significance level of 0.05 with a two-sided alternative.

Descriptive statistics were determined for baseline maternal characteristics, GWG, GDM diagnosis and OGTT glucose parameters, partitioned by time period. Means ± standard deviation (SD) for normally distributed data and medians with interquartile range (IQR) for non-normally distributed data, proportions and counts were calculated. Differences in the samples between cohorts were assessed using one-way analysis of variance and chi-squared test or Fisher’s exact test for continuous and categorical data, respectively.

The primary outcome was assessed using univariate and multivariate generalised estimating equations (GEE) models. Models were equipped with a logit link function. Robust covariance estimates with an AR(1) correlation structure were used. Multivariate GEE models were adjusted for maternal baseline characteristics (age, BMI, ethnicity, socioeconomic disadvantage, nulliparity, prior history of GDM, family history of diabetes and PCOS) and GWG at each antenatal appointment. Odds ratios (ORs), 95% confidence intervals and p-values were reported. Population attributable fractions (PAFs) were computed using Levin’s formula for maternal characteristics on GDM diagnoses using adjusted ORs.

## Results

**Fig. 1:**
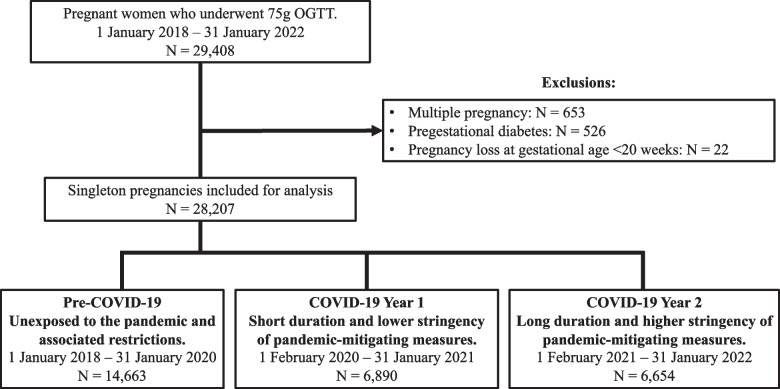
Study flowchart. OGTT: oral glucose tolerance test

Between 1^st^ January 2018 and 31^st^ January 2022, 28,207 pregnancies met the inclusion criteria. 14,663 pregnancies were included in the pre-COVID-19 cohort, 6,890 in the COVID-19 Year 1 cohort and 6,654 in the COVID-19 Year 2 cohort, representing 76.0%, 75.2% and 75.8% of all pregnancies in the three respective time periods (Fig. 1).

### Maternal characteristics

Baseline maternal characteristics for the pre-COVID-19 and two pandemic exposure periods are presented in Table 1. Maternal age at conception (30.7 ± 5.0 years pre-COVID-19 vs 31.0 ± 5.0 years in COVID-19 Year 1 vs 31.3 ± 5.0 years in COVID-19 Year 2; *p* < 0.001), pre-pregnancy BMI (25.5 ± 5.7 kg/m^2^ vs 25.7 ± 5.6 kg/m^2^ vs 26.1 ± 5.7 kg/m^2^; *p* < 0.001) and proportion of obese women (17.5% vs 18.1% vs 20.7%; *p* < 0.001) increased across exposure periods. All cohorts were ethnically and socioeconomically diverse and there were progressive shifts in ethnic profile characterised by rising proportions of South Asian and Caucasian ethnicities. The proportion of nulliparous pregnancies decreased, whereas there were rising proportions with prior history of GDM, family history of diabetes and PCOS. COVID-19 infection rate in pregnancy increased in COVID-19 Year 2 but remained low. Baseline maternal characteristics were compared between pregnancies meeting the inclusion criteria and those missing OGTT results for each cohort. Women missing OGTT results were older and less likely to have a prior history of GDM, but these differences were consistently observed for all three time periods. There were no differences in other baseline characteristics.

**Table 1:** Baseline maternal characteristics and gestational weight gain of the pre-COVID-19 cohort and cohorts with pandemic exposure

Characteristics	Two Years Pre-COVID-19 *N* = 14,663	COVID-19 Year 1 *N* = 6,890	COVID-19 Year 2 *N* = 6,654	*P*-value
Maternal age at conception				< 0.001
< 20 years	158 (1.1%)	67 (1.0%)	62 (0.9%)	
20–24.9 years	1,022 (7.0%)	418 (6.1%)	386 (5.8%)	
25–34.9 years	9,085 (62.0%)	4,322 (62.7%)	3,980 (59.8%)	
35–39.9 years	3,857 (26.3%)	1,803 (26.2%)	1,924 (28.9%)	
40 years and over	541 (3.7%)	280 (4.1%)	302 (4.5%)	
Pre-pregnancy BMI category^a^				< 0.001
Underweight	637 (4.3%)	267 (3.9%)	209 (3.1%)	
Healthy weight	7.453 (50.8%)	3,462 (50.3%)	3,100 (46.6%)	
Overweight	4,006 (27.3%)	1,915 (27.8%)	1,971 (29.6%)	
Obese	2,567 (17.5%)	1,246 (18.1%)	1,374 (20.7%)	
Ethnicity				< 0.001
South Asian	4,021 (29.9%)	2,087 (30.9%)	2,153 (33.0%)	
Middle Eastern	5,088 (37.3%)	2,396 (35.4%)	1,949 (29.8%)	
Caucasian	2,249 (16.7%)	1,274 (18.8%)	1,397 (21.4%)	
East and Southeast Asian	1,963 (14.6%)	899 (13.3%)	896 (13.7%)	
Aboriginal and Torres Strait Islander	205 (1.5%)	109 (1.6%)	140 (2.1%)	
Socioeconomic disadvantage: SEIFA quintile^b^				0.001
Q1 (most disadvantaged)	2,465 (23.5%)	1,122 (22.3%)	1,024 (21.5%)	
Q2	2,785 (26.6%)	1,397 (27.7%)	1,350 (28.4%)	
Q3	1,820 (17.4%)	823 (16.3%)	814 (17.1%)	
Q4	1,465 (14.0%)	797 (15.8%)	745 (15.7%)	
Q5 (least disadvantaged)	1,936 (18.5%)	901 (17.9%)	827 (17.4%)	
Smoking in pregnancy	699 (4.8%)	314 (4.6%)	276 (4.2%)	0.20
Nulliparity	6,627 (45.2%)	3,013 (43.7%)	2,649 (39.8%)	< 0.001
Prior history of gestational diabetes	962 (6.6%)	511 (7.4%)	565 (8.5%)	< 0.001
First degree family member or sister with diabetes	5,769 (39.7%)	2,770 (40.5%)	2,904 (44.2%)	< 0.001
Polycystic ovarian syndrome	917 (6.3%)	492 (7.1%)	466 (7.0%)	0.02
History of mental health conditions	1,814 (12.4%)	1,006 (14.6%)	952 (14.3%)	< 0.001
COVID-19 infection in pregnancy	0	8 (0.1%)	111 (1.7%)	< 0.001
Rate of 1^st^ and 2^nd^ trimester gestational weight gain (kg/week)	0.59 ± 0.58	0.64 ± 0.61	0.64 ± 0.62	< 0.001
Excessive 2^nd^ trimester gestational weight gain by IOM^c^	7,015 (64.3%)	3,658 (66.0%)	3,577 (66.6%)	0.009

^a^BMI Body mass index in kg/m^2^

^b^SEIFA Socio-Economic Indexes for Areas 2016 [16]

^c^IOM Institute of Medicine and National Research Council recommendations for gestational weight gain 2009 [19]

### Gestational weight gain

The number of weight measurements performed per pregnancy decreased (7.5 ± 2.5 vs 7.4 ± 2.4 vs 7.0 ± 2.3; *p* < 0.001). As shown in Table 1, the rate of GWG in first and second trimester (0.59 ± 0.58 kg/week vs 0.64 ± 0.61 kg/week vs 0.64 ± 0.62 kg/week; *p* < 0.001) and proportion exceeding recommended second trimester GWG (64.3% vs 66.0% vs 66.6%; *p* = 0.009) increased during the pandemic.

### Gestational diabetes

**Fig. 2:**
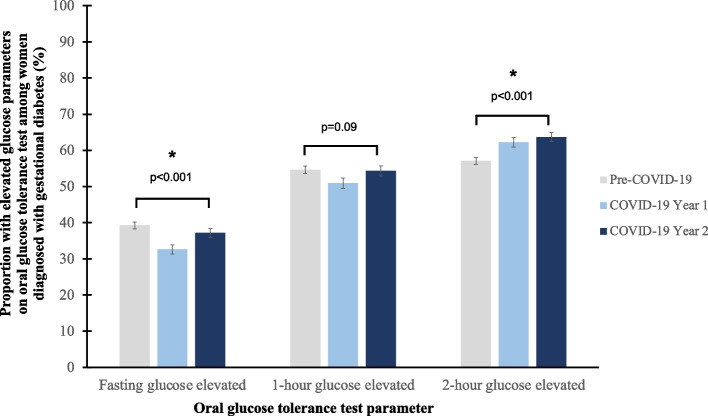
Proportion of women, of those diagnosed with gestational diabetes in accordance with the International Association of Diabetes and Pregnancy Study Groups (IADPSG) criteria, exceeding fasting glucose, 1-h glucose and 2-h glucose cut-offs on oral glucose tolerance test pre-COVID-19 and during each pandemic exposure period [20]

The proportion of women meeting OGTT criteria for GDM increased across exposure periods (21.2% vs 22.9% vs 24.8%; *p* < 0.001, Table 2). Table 2 also displays the mean fasting, 1-h and 2-h OGTT glucose values for pregnancies complicated by GDM. Of those diagnosed with GDM, proportions exceeding fasting, 1-h and 2-h normal cut-offs are presented in Fig. 2, showing rising proportion diagnosed with elevated 2-h glucose.

**Table 2:** Proportion of pre-COVID-19 and pandemic exposure cohorts diagnosed with gestational diabetes and their oral glucose tolerance test glucose values

Outcome	Two Years Pre-COVID-19	COVID-19 Year 1	COVID-19 Year 2	*P*-value
**Gestational diabetes**	3,114 / 14,663 (21.2%)	1,580 / 6,890 (22.9%)	1,649 / 6,654 (24.8%)	< 0.001
**Oral glucose tolerance test results for women meeting IADPSG criteria for gestational diabetes (mmol/L)**^**a**^	*N* = 3,1224	*N* = 1,580	*N* = 1,649	
**Fasting glucose**	4.9 ± 0.7	4.8 ± 0.7	4.9 ± 0.7	0.004
**1-h glucose**	9.7 ± 2.00	9.5 ± 2.1	9.7 ± 2.1	0.001
**2-h glucose**	8.4 ± 1.8	8.6 ± 1.8	8.7 ± 1.9	< 0.001

^a^IADPSG International Association of Diabetes and Pregnancy Study Groups [20]

Both pandemic periods were associated with increased GDM risk compared to pre-COVID-19 on univariate analysis (OR 1.11 [1.02, 1.21], *p* = 0.02 for COVID-19 Year 1 and OR 1.34 [1.23, 1.46], *p* < 0.001 for COVID-19 Year 2). Baseline maternal characteristics such as older age, overweight and obesity, non-Caucasian ethnicity, nulliparity, prior history of GDM, family history of diabetes and PCOS were associated with increased GDM (Table 3). COVID-19 infection was not associated (OR 0.66 [0.37, 1.17], *p* = 0.2). First and second trimester GWG was not associated with GDM on univariate analysis but GDM risk increased by 31% per 1 kg per week increase in GWG after adjusting for baseline characteristics such as BMI (OR 1.31 [1.15, 1.49], *p* < 0.001). After adjusting for baseline characteristics and GWG, COVID-19 Year 2, but not COVID-19 Year 1, remained significantly associated with increased risk of GDM (OR 1.17 [1.06, 1.28], *p* = 0.01).

**Table 3:** Unadjusted and adjusted odds ratios for the risk of gestational diabetes

Characteristics	Univariate OR (95% CI)^a^	*P*-value	Multivariate OR (95% CI)^a^	*P*-value
Exposure period				
Pre-COVID-19	Reference			
COVID-19 Year 1	1.11 (1.02, 1.21)	0.02	1.05 (0.96, 1.15)	0.33
COVID-19 Year 2	1.34 (1.23, 1.46)	< 0.001	1.17 (1.06, 1.28)	0.01
Rate of 1^st^ and 2^nd^ trimester gestational weight gain (kg/week)	1.07 (0.95, 1.21)	0.3	1.31 (1.15, 1.49)	< 0.001
Maternal age at conception				
< 20 years	0.34 (0.20, 0.56)	< 0.001	0.39 (0.23, 0.66)	< 0.001
20–24.9 years	0.49 (0.41, 0.59)	< 0.001	0.54 (0.44, 0.65)	< 0.001
25–34.9 years	Reference			
35–39.9 years	1.64 (1.52, 1.78)	< 0.001	1.57 (1.44, 1.71)	< 0.001
40 years and over	1.96 (1.66, 2.32)	< 0.001	1.92 (1.59, 2.31)	< 0.001
Pre-pregnancy BMI category^b^				
Underweight	0.79 (0.64, 0.97)	0.03	0.84 (0.68, 1.05)	0.12
Healthy weight	Reference			
Overweight	1.59 (1.46, 1.72)	< 0.001	1.61 (1.48, 1.76)	< 0.001
Obese	1.86 (1.70, 2.05)	< 0.001	2.10 (1.89, 2.33)	< 0.001
Ethnicity				
Caucasian	Reference			
South Asian	2.27 (2.02, 2.55)	< 0.001	2.24 (1.98, 2.53)	< 0.001
Middle Eastern	1.54 (1.36, 1.73)	< 0.001	1.50 (1.32, 1.70)	< 0.001
East and Southeast Asian	1.95 (1.70, 2.23)	< 0.001	2.14 (1.85, 2.47)	< 0.001
Aboriginal and Torres Strait Islander	1.42 (1.04, 1.94)	0.03	1.47 (1.05, 2.05)	0.03
Socioeconomic disadvantage: SEIFA quintile^c^				
Q1 (most disadvantaged)	1.20 (1.05, 1.38)	0.01	1.31 (1.13, 1.51)	< 0.001
Q2	1.16 (1.02, 1.320	0.02	1.06 (0.92, 1.22)	0.41
Q3	1.10 (0.95, 1.270	0.21	1.01 (0.87, 1.18)	0.88
Q4	0.99 (0.85, 1.16)	0.90	0.85 (0.72, 1.00)	0.05
Q5 (least disadvantaged)	Reference			
Nulliparity	0.98 (0.92, 1.06)	0.66	1.60 (1.48, 1.74)	< 0.001
Prior history of gestational diabetes	6.25 (5.54, 7.06)	< 0.001	6.05 (5.28, 6.92)	< 0.001
First degree family member or sister with diabetes	1.73 (1.62, 1.86)	< 0.001	1.40 (1.29, 1.50)	< 0.001
Polycystic ovarian syndrome	1.60 (1.41, 1.83)	< 0.001	1.33 (1.15, 1.53)	< 0.001

Odds ratios for COVID-19 pandemic period on risk of gestational diabetes in multivariate model adjusted for maternal age, pre-pregnancy body mass index, ethnicity, socioeconomic disadvantage, nulliparity, prior history of gestational diabetes, family history of diabetes, history of polycystic ovarian syndrome and first and second trimester gestational weight gain

^a^OR Odds ratio, CI Confidence interval

^b^BMI Body mass index in kg/m^2^

^c^SEIFA Socio-Economic Indexes for Areas 2016 [16]

To estimate contributions of each baseline maternal characteristic to GDM, PAFs were estimated using adjusted ORs (Table 4). PAFs were highest for pre-pregnancy BMI (25% of GDM attributable to overweight and obesity), ethnicity (21% attributable to South Asian ethnicity), maternal age (16% attributable to age ≥ 35 years), prior history of GDM (16%) and nulliparity (16%).

**Table 4:** Proportion of gestational diabetes attributable to each maternal characteristic

Characteristics	Population attributable fraction (PAF) (95% CI)^a^
Maternal age at conception	
< 20 years	-0.01 (-0.02, 0.00)
20–24.9 years	-0.03 (-0.05, -0.02)
25–34.9 years	Reference
35–39.9 years	0.13 (0.11, 0.15)
40 years and over	0.03 (0.02, 0.04)
Pre-pregnancy BMI category^b^	
Underweight	-0.01 (-0.01, 0.00)
Healthy weight	Reference
Overweight	0.12 (0.11, 0.14)
Obese	0.13 (0.11, 0.14)
Ethnicity	
Caucasian	Reference
South Asian	0.21 (0.19, 0.23)
Middle Eastern	0.10 (0.07, 0.13)
East and Southeast Asian	0.08 (0.07, 0.09)
Aboriginal and Torres Strait Islander	0 (0.00, 0.01)
Socioeconomic disadvantage: SEIFA quintile^c^	
Q1 (most disadvantaged)	0.04 (0.02, 0.06)
Q2	0.01 (-0.02, 0.03)
Q3	0 (-0.02, 0.02)
Q4	-0.02 (-0.04, 0.00)
Q5 (least disadvantaged)	Reference
Nulliparity	0.16 (0.14, 0.19)
Prior history of gestational diabetes	0.16 (0.15, 0.16)
First degree family member or sister with diabetes	0.14 (0.11, 0.16)
Polycystic ovarian syndrome	0.02 (0.01, 0.03)

^a^CI Confidence interval

^b^BMI Body mass index in kg/m^2^

^c^SEIFA Socio-Economic Indexes for Areas 2016 [16]

## Discussion

This multicentre cohort study of GDM pre-COVID-19 and during the first and second years of the pandemic demonstrated a progressive increase in the rate of GDM with pandemic exposure. The pandemic was associated with changes in maternal baseline characteristics, including increases in most of the traditional risk factors for GDM, and greater GWG. Adjusting for these factors attenuated the effect of pandemic exposure on GDM risk, but the second year of the pandemic, characterised by longer duration and higher stringency of pandemic-related community restrictions, remained independently associated with increased GDM diagnosis.

The findings of this study add to the growing body of observational evidence of increased GDM risk associated with exposure to the COVID-19 pandemic. Studies on the risk of GDM, mostly from countries with high case burden during the study periods, found significantly higher rates of GDM in 2020 compared to pre-pandemic [1, 3]. Other research on overall pregnancy outcomes have reported inconsistent findings, with some supporting an increased risk and others finding no difference in GDM with COVID-19 lockdown, possibly due to heterogeneity of study populations and GDM diagnostic testing recommendations [4, 21–23].

Analysis of baseline maternal characteristics revealed progressive changes with pandemic exposure. The pandemic may have affected both the general population and specifically the subset of women conceiving. Gradual shifts in the underlying population independent of pandemic exposure are also possible, but the study compared cohorts in directly adjacent timeframes. Maternal age increased, which may reflect an indirect effect of international border closures and reduced migration into the population [10]. COVID-19-related concerns may have also discouraged younger women with lower pressures of fertility considerations from conceiving during the pandemic. There were increasing proportions of pregnant women of South Asian and Caucasian ethnicities, whereas women of Middle Eastern ethnicity decreased. These shifts in ethnic profile may have contributed to the rising proportion of women diagnosed with GDM due to elevated 2-h glucose (Fig. 2) [24]. Rising proportion of Caucasian women may be a consequence of international border closures. The reasons underlying the opposing trends for South Asian and Middle Eastern ethnicities are unclear, but may suggest cultural differences affecting response to the pandemic and reduced social supports, rather than changes in migration pattern given border restrictions [25].

Lifestyle changes due to working remotely, home schooling, gym closures, periodic lockdowns and fear of exposure to COVID-19 infection may have contributed to both increasing pre-pregnancy BMI and GWG [25]. Surveys on pregnant women with GDM have revealed increase in sedentary behaviour during the COVID-19 pandemic but more data evaluating lifestyle and cultural factors in ethnically diverse populations are needed [26]. The efficacy of dietetic services delivered by telehealth compared to traditional models of care and potential implications for GWG are also unknown.

We found that exposure to COVID-19 Year 1 was no longer significant and the association of COVID-19 Year 2 with GDM was attenuated after adjusting for baseline maternal characteristics and GWG, suggesting that the shifts in maternal sociodemographic profile partly account for the increased risk. Many of the changes were to traditional GDM risk factors, our analyses corroborating the contribution of obesity, South Asian ethnicity, older age and prior history of GDM in particular, all of which increased with pandemic exposure. GWG was only significant on multivariate analysis, likely due to negative confounding by pre-pregnancy BMI.

However, the persistence of a significant association between exposure to COVID-19 Year 2 and GDM after adjustment for maternal characteristics and GWG suggests that there were unmeasured factors increasing the risk of GDM for pregnant women during the second year of the pandemic. The second year was characterised by prolonged and highly stringent community restrictions. Limitations to physical activity may have had consequences not entirely accounted for by adjusting for BMI and GWG, such as adverse effects on insulin sensitivity [27]. Psychological stress has previously been shown to be associated with risk of GDM, and pandemic-related stress, potentially heightened in the second year due to pandemic duration, rising cases and escalating restrictions, and potential effect on inflammatory processes, has been proposed as a mechanism of increased incidence during the COVID-19 pandemic [1, 28]. Validated scales assessing perceived stress and measurement of inflammatory markers would be useful for exploring this hypothesis.

A major strength of our study is that it comprises a large, sociodemographically diverse population experiencing different types and intensity of exposures across time periods, facilitating inferences about possible effects of pandemic-mitigating measures. Although multicentre, antenatal care and COVID-19-related healthcare changes were governed by one health district and were thus consistent. We had access to highly complete demographic data and detailed information on antenatal weights, strengthening our analysis of GWG, as well as information about maternal COVID-19 infection to confirm low maternal infection rates.

A limitation is that although universal diagnostic testing for GDM with 75 g OGTT was recommended, some women did not undergo an OGTT, with OGTT results missing for over 20% of pregnancies. This may have been due to inability to tolerate OGTT, contraindications or use of alternative GDM diagnostic methods (which were not accepted for inclusion in this study), but the reasons for OGTT omission were not available. Meaningful reduction in OGTTs during the pandemic periods is unlikely given the proportions of missing OGTTs for each cohort and the expected effect on the primary outcome, if any, of decrease in performance of OGTT during the pandemic exposure periods, for example due to need to isolate for infection or close contact status, would be more missed diagnoses and thus underestimation of GDM risk. Other studies assessing the risk of GDM during COVID-19 also only included women who underwent 75 g OGTT, [1–3], and a risk factor-based approach rather than universal diagnostic testing is recommended in some centers [2]. The analysis did not take into account specific exposure timing and duration for each individual pregnancy, as this data was not prospectively collected and population level restrictions may not be applicable at an individual level. As cumulative exposure during pregnancy and exposure to lockdown in first trimester may particularly affect GDM risk, a greater association with GDM may have been observed if individual level exposure to stringent measures was able to be analysed and the study population limited to women with prolonged first trimester exposure [1, 3]. Detailed dietary and physical activity data and validated measures of anxiety and stress were also not available, and thus changes in these potential mediators across time periods could not be quantified.

In conclusion, we found a progressive increase in the risk of GDM during the COVID-19 pandemic. Changes in maternal sociodemographic profile, BMI and GWG were potential contributors to the increased rate of GDM, but exposure to the second year of the pandemic and its stringent pandemic-mitigating restrictions remained independently associated after adjusting for these factors. These findings highlight the importance of developing public health initiatives to limit the impact of current and future pandemics and pandemic-mitigating measures on modifiable risk factors, as well as the need for further research to explore unrecognised mediators of increased risk.

## Supplementary Information

Additional file 1: **Supplementary Table 1. **Chronology of COVID-19-mitigating communitymeasures affecting the study population [13]

## Data Availability

The data generated during and/or analysed during the current study are available from the corresponding author on reasonable request.
