# Supplementary material for: Indirect effects of the COVID-19 pandemic on risk of gestational diabetes and factors contributing to increased risk in a multiethnic population: a retrospective cohort study
Source: BMC Pregnancy Childbirth. 2023 May 12;23:341. doi: 10.1186/s12884-023-05659-6 (PMC10175922; doi:10.1186/s12884-023-05659-6)
Supplement: Supplementary file 1 — Supplementary Table 1. Chronology of COVID-19-mitigating communitymeasures affecting the study population [13] [file 12884_2023_5659_MOESM1_ESM.docx]

**Supplementary Information**

**Supplementary Table 1: Chronology of COVID-19-mitigating community measures affecting the study population[13]**

| Timeframe | Type of Measure | Details of COVID-19 Mitigation Measure |
| --- | --- | --- |
| March 2020 | Social gathering | Cancellation of events with >500 people  Ban on indoor gatherings >100 people  Social distancing requirement of 1.5m |
|  | Business | Closure of non-essential businesses |
|  | Border and travel | Escalating travel restrictions:  Quarantine for all international arrivals  Travel advice raised to “Do not travel overseas”  International border closure to all non-citizens and residents |
| March 2020 – May 2020 | General public lockdown | General public stay at home order  Public gatherings limited to two persons |
| May 2020 | Social gathering | Relaxation of restrictions to public gatherings, household visitors, community sport |
|  | Education | Return to face-to-face teaching |
| June 2020 | Border and travel | Permission to travel to regional NSW |
| July 2020 | Border and travel | Reinstatement of interstate border restrictions (due to rising cases in Victoria) |
| October 2020 | Border and travel | New Zealand travel bubble: Quarantine not required for travel |
| December 2020 | Area-specific restrictions | Localised stay at home orders for areas with outbreaks |
| June 2021 –October 2021 | General public lockdown | General public stay at home order  Outdoor exercise limited to within their local area or within 10km of home and to two persons  Shopping for essential goods and services by one person only  Ban on entry into Greater Sydney |
| July 2021 | Area-specific restrictions in addition to general public lockdown | Increased stringency of lockdown for study population:  Outdoor exercise and shopping limited to within 5km of home |
| August 2021 | Area-specific restrictions in addition to general public lockdown | Further increased stringency of lockdown for study population:  Curfews from 9pm-5am  Outdoor exercise limited to one hour per day  Closure of retail except for click and collect services |
| October 2021 | Social gathering | Lifting of lockdown and stepwise relaxation of limitations for vaccinated people |
|  | Education | Return to face-to-face teaching |
| November 2021 | Border and travel | International border restrictions eased for the vaccinated |
| December 2021 | Social gathering | Relaxation of restrictions for unvaccinated people  Removal of density limits for public gatherings |
